# Simulation of Human Small Intestinal Digestion of Starch Using an In Vitro System Based on a Dialysis Membrane Process

**DOI:** 10.3390/foods9070913

**Published:** 2020-07-10

**Authors:** Carol González, Daniela González, Rommy N. Zúñiga, Humberto Estay, Elizabeth Troncoso

**Affiliations:** 1 Department of Chemistry, Universidad Tecnológica Metropolitana, Las Palmeras 3360, Ñuñoa, Santiago 7800003, Chile; carol.gonzalezm@utem.cl (C.G.); gonzalezdelcarpio@gmail.com (D.G.); 2 Department of Biotechnology, Universidad Tecnológica Metropolitana, Las Palmeras 3360, Ñuñoa, Santiago 7800003, Chile; rommy.zuniga@utem.cl; 3 Programa Institucional de Fomento a la Investigación, Desarrollo e Innovación, Universidad Tecnológica Metropolitana, Ignacio Valdivieso 2409, San Joaquín, Santiago 8940577, Chile; 4 Advanced Mining Technology Center (AMTC), University of Chile, Av. Tupper 2007, AMTC Building, Santiago 8370451, Chile; humberto.estay@amtc.cl

**Keywords:** starch, in vitro digestion, human small intestine, nutrient absorption, mass transfer

## Abstract

This work deepens our understanding of starch digestion and the consequent absorption of hydrolytic products generated in the human small intestine. Gelatinized starch dispersions were digested with α-amylase in an in vitro intestinal digestion system (*i*-IDS) based on a dialysis membrane process. This study innovates with respect to the existing literature, because it considers the impact of simultaneous digestion and absorption processes occurring during the intestinal digestion of starchy foods and adopts phenomenological models that deal in a more realistic manner with the behavior found in the small intestine. Operating the *i*-IDS at different flow/dialysate flow ratios resulted in distinct generation and transfer curves of reducing sugars mass. This indicates that the operating conditions affected the mass transfer by diffusion and convection. However, the transfer process was also affected by membrane fouling, a dynamic phenomenon that occurred in the *i*-IDS. The experimental results were extrapolated to the human small intestine, where the times reached to transfer the hydrolytic products ranged between 30 and 64 min, according to the flow ratio used. We consider that the *i*-IDS is a versatile system that can be used for assessing and/or comparing digestion and absorption behaviors of different starch-based food matrices as found in the human small intestine, but the formation and interpretation of membrane fouling requires further studies for a better understanding at physiological level. In addition, further studies with the *i*-IDS are required if food matrices based on fat, proteins or more complex carbohydrates are of interest for testing. Moreover, a next improvement step of the *i-*IDS must include the simulation of some physiological events (e.g., electrolytes addition, enzyme activities, bile, dilution and pH) occurring in the human small intestine, in order to improve the comparison with in vivo data.

## 1. Introduction

### 1.1. In Vitro Systems to Simulate Human Digestion

Evidence has shown that nutrition, and in particular postprandial glycaemia, plays a crucial role in the development of type-2 diabetes. Therefore, the study and understanding of human digestion has turned out to be of great interest to predict and control the absorption of glucose from the intake of foods with high starch content [1,2,3]. Human digestion is a complex process in which mechanical and enzymatic transformation of food macromolecules take place simultaneously and hydrolytic products are absorbed [4,5]. Most of the systems developed for food disintegration and nutrient release research are made as static in vitro systems. These consist of placing the food in a series of reactors or bioreactors which recreate the physicochemical and enzymatic environment of each digestive compartment (e.g., mouth, stomach and small intestine) [5]. However, these systems simplify the behavior of the human gastrointestinal tract and are unrealistic, because they cannot simulate the real physiological processes, such as mixing, peristaltic movements, injection in real time of digestive enzymes or changes in pH conditions over time [4,5,6]. Now, among the intestinal experimental absorption models, in vivo models are used to study nutrient or drug bioavailability when food or drug formulations are administered orally, or directly into the intestine, in humans or suitable animal species. These in vivo models are the most accurate way to determine intestinal permeability, fraction absorbed and bioavailability, because the impacts of different physiological parameters are considered [7,8]. Among the techniques used for these kind of in vivo studies, we highlight (i) intestinal perfusion to assess the effective intestinal permeability of phytochemicals, sulforaphane, quercetin-3,4′-glucoside [9], vitamin E and gallic acid [10] and drugs such as tripterine [11] and amoxicillin [12] or drugs that are incompletely absorbed in the proximal small intestine [13] and (ii) ileostomy to determine the bioavailability of folic acid [14], amoxicillin [15], sugars [16] and (poly)phenols [17]. However, in vivo models have ethical, economic and technical restrictions that limit their application. In addition, in vivo models are less applicable for phenomenological understanding of the individual impacts of physiological factors on nutrient bioavailability [7]. For this reason, a real need exists for the development of in vitro models that simulate the real physiological processes that occur in the human gastrointestinal tract during the digestion of food, which must be flexible, precise, controlled and reproducible [4,6]. Therefore, in recent years, there has been a development of dynamic in vitro models capable of simulating the physiology of the stomach and small intestine, which can predict in vivo behavior, such as decomposition of solids and nutrient absorption. These models can be used as a valuable research tool for the study and understanding of changes and interactions as well as the bioaccessibility and bioavailability of nutrients, drugs and non-nutrient compounds [5,6,18].

The study of in vitro hydrolysis of starch has been of great interest because it can be associated with the glycemic response, which is an indicator of the postprandial glucose response for foods with high starch content [19,20,21,22]. Glucose causes metabolic stress when it is present in high levels in the blood. High glucose concentration is associated with an increased risk of suffering from some diseases, such as type-2 diabetes, cancer and cardiovascular disease [23]. The realization of in vitro studies to determine the rate of starch digestion and absorption has advantages over in vivo studies, because it is much more economical and does not require ethical authorization. To simulate in vitro absorption, artificial or biological membrane systems or assays based on biological cell monolayers have been used [24,25,26]. For in vitro intestinal systems, semipermeable membranes are often used, which act as a selective barrier to keep the enzymes separated from their digestion products, avoiding the inhibition of enzymatic hydrolysis [18]. Recently, Dupont et al. [5] reviewed the validation of eight dynamic in vitro models against in vivo data, namely DGM, HGS, ARCOL, DIDGI^®^, TIM, SHIME^®^, ESIN and simgi^®^. Of the eight models, two of them simulate the stomach (DGM and HGS), one simulates the large intestine (ARCOL) and the other five simulate the stomach and small intestine. Of the five models that simulate the small intestine, two do not take into account intestinal nutrient absorption (DIDGI^®^ and simgi^®^). The other three models use membranes to simulate intestinal absorption (TIM, SHIME^®^ and ESIN). Specifically, the TIM and ESIN models use a dialysis hollow fiber membrane to mimic the passive absorption of water and hydrolytic products from digestion in the small intestine. A description of other more established models based on membrane systems can be found in Gim et al. [18], where the advantages and limitations of existing approaches are briefly discussed. For example, in 2010, the in vitro small intestine model (SIM) [27] was fabricated to study glucose absorption using semi-permeable dialysis membranes, also mimicking intestinal peristalsis and segmentation motion; however, fluids secretion, pH and body temperature were not considered. In 2014, the SIM was improved by the dynamic duodenum (DDuo) model, which incorporated simulated intestinal secretions and pressure zones mimicking segmentations and peristalsis [28]. After two years, the human duodenal model (HDM) was proposed to estimate nutrient absorption using a membrane dialysis and to simulate peristaltic motion using pneumatic movement, which also incorporated the sigmoidal shape of the first portion of the human small intestine [29]. In spite of all the efforts to simulate intestinal digestion and absorption effectively, the complex phenomenology of intestinal mass transfer is still a challenging problem.

The use of hollow fiber membranes for the dialysis process is common practice in studies focused on hemodialysis. These kinds of membranes function as an artificial kidney, in which the membrane acts as a separation barrier for metabolic wastes [30]. During the dialysis process, the driving force is the difference in concentration of the compounds, between the lumen and the shell side of the membrane. For hemodialysis, the phenomenology of mass transfer is promoted using a diffusion process [30,31,32,33,34,35] and a convective effect, which is determined by a pressure difference between the lumen and shell side. This pressure difference generates a water transfer or solution, as described elsewhere in the literature [24,25,33]. Transmembrane pressure is the driving force for other membrane processes, such as micro-, ultra- and nanofiltration processes. The dialysis process is the only membrane process capable of transferring solutes with high molecular weight by diffusive mass transfer, due to a concentration difference. Considering this last point, in a previous work performed by our research team [18], we fabricated an in vitro small intestine model (*i*-IDS) where a dialysis membrane system was used as an artificial small intestine, due to its ability to simulate the phenomena of diffusive mass transfer and absorption occurring in the small intestine and the arrangement of the large transfer area of the membrane module. The system allowed glucose transfer and absorption to be studied. We then proposed mathematical models to describe the diffusive and convective mechanisms involved.

### 1.2. Kinetics of Starch Digestion

Starch is the main source of digestible carbohydrates that contributes significantly to total energy intake, and it is present in foods such as cereals, corn, rice, wheat and potatoes [23,36]. Amylose and amylopectin correspond to the two main polymers that compose the starch granule [37]. These polymers contain glucose bound by α-1,4-glycosidic bonds, which can be hydrolyzed by the α-amylase enzyme to produce short-chain linear oligosaccharides. The ramifications of amylopectin are formed through α-1,6 glycosidic bonds which are resistant to the action of α-amylase [38]. The starch granule has a semi-crystalline structure which, in the presence of water and temperature above 58 °C, undergoes a structural change called gelatinization [36]. Starch gelatinization consists of the swelling of the starch granules, as a consequence of water absorption, increasing the availability of starch for enzymatic hydrolysis by digestive enzymes [39,40,41,42]. The physical state of the starch will determine the accessibility of the digestive enzymes. There are different states in which starch can be present in foods, namely (i) native, where digestibility is low, (ii) gelatinized, where digestibility is high or (iii) retrograded, where the digestibility is intermediate between native and gelatinized [3]. Retrogradation is the phenomenon occurring when the molecules comprising gelatinized starch begin to re-associate in an ordered structure [36]. The hydrolysis of starch consists in the breaking down of starch polymers into short chain fragments, such as glucose monomers, maltose and dextrins, where most of the hydrolysis of starch is carried out by the pancreatic α-amylase in the small intestine [19,40]. This process occurs during digestion in order to obtain monosaccharides that are capable of being absorbed through the walls of the small intestine and are subsequently transported by the bloodstream to the cells to be used as an energy source for different functions of the human body. In several studies, the hydrolysis of starch has been modeled by first-order reaction kinetics [21,43,44,45], obtaining information about the degradation rate constant and the amount of product generated. This information is very valuable for the generation of phenomenological models for enzymatic starch hydrolysis and absorption during in vitro gastrointestinal digestion.

**Figure 1:**
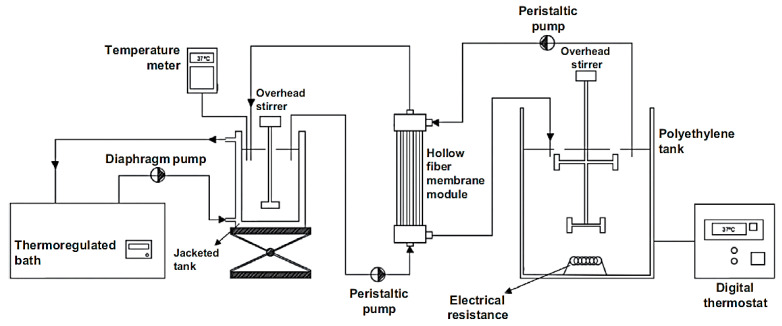
Experimental diagram of the *i*-IDS to simulate starch digestion and mass transfer of reducing sugars in the human small intestine [18].

Based on the above, the objective of this work is to deepen our previous study [18], using the same in vitro digestion system, namely the *i*-IDS (Figure 1). In this system, a dialysis membrane was used to simulate the absorption process of glucose molecules in the small intestine. In the present study, we take on the challenge of simulating a more realistic phenomenon of the human intestinal digestion. Starting with the use of a food matrix, such as gelatinized potato starch, and using an enzyme for its degradation, we study the absorption process of the hydrolytic products generated by means of a dialysis membrane, obtaining information on the phenomenological model of generation and transfer of sugars. With these results we expect to obtain a more realistic understanding of the functioning of the digestion and absorption processes in the small intestine to achieve a simulation that resembles the operation of this organ and to reproduce and scale-up the results to human digestion.

## 2. Materials and Methods

### 2.1. Materials

Commercial potato starch (Chuño Delicado, Chile) purchased from a local market (Table 1) was used as a model starch-based food. α-amylase (BAN 480L, Novozymes Company, Frederiksberg, Denmark), produced by submerged fermentation of a selected strain of *Bacillus amyloliquefaciens*, was kindly provided by Blumos Chile (Santiago, Chile). BAN 480L is available with a standard strength of 480 KNU/g and relative activity at 37 °C of 67% and 92% at pH 7, as provided by the manufacturer. Monobasic sodium phosphate, dibasic sodium phosphate (Sigma-Aldrich, St. Louis, MO, USA) and sodium azide (Merck, Darmstadt, Germany) were used for the preparation of phosphate buffer. For the DNS method, 3,5-Dinitrosalicylic acid, crystalline phenol and sodium bisulfite (Acros Organics, Geel, Belgium), sodium potassium tartrate were used. In addition, solutions of sodium hydroxide and hydrochloric acid (Fisher Scientific, Waltham, MA, USA) were used to adjust the pH of samples by means of manual titration. Ultra-purified water (resistivity 15.0 MΩ-cm) was used for the preparation of the starch dispersions.

### 2.2. Preparation of Gelatinized Starch Dispersions

The starch dispersion consisted of potato starch (1% *w/v*) and phosphate buffer. The choice of this concentration ensured a Newtonian behavior of the gelatinized starch dispersion, which had a viscosity of 0.0165 ± 0.0020 Pa·s (data not shown). For the samples, it was necessary to maintain a pH equal to 7.0, simulating the conditions of the small intestine. For the preparation of 1 L of phosphate buffer the following was used: 5.421 g of monobasic sodium phosphate, 14.288 g of dibasic sodium phosphate and 0.2 g of sodium azide. For the study, 2 L of starch dispersion were prepared in a 3 L double-walled vessel, then the dispersion was stirred. Prior to the assays, water from a thermoregulated bath was circulated at 75 °C inside the walls of the vessel for 1 h to ensure the gelatinization of the starch at a temperature of 65 °C, considering the heat loss through the walls of the vessel towards the ambient temperature.

The gelatinization temperature of the starch was determined using differential scanning calorimetry equipment (DSC 1 STARe System, Mettler Toledo, Zurich, Switzerland), coupled to an immersion cooler (TC100, Huber, Offenbach). The gelatinization temperature was evaluated measuring the endothermic transition enthalpy of starch suspensions. Measurements were performed in aluminum pans (~100 μL of sample) sealed under manual pressure and reweighted after the process in order to check that no significant evaporation occurred during the measurement. An aluminum pan filled with pure water was used as reference. To assess the reversibility of the heat effects on the samples, a cooling step was performed, followed by a re-heating interval. The heating profiles were conducted from 25 to 95 °C at a heating rate of 5 °C/min followed by a cooling step from 95 to 25 °C with a 40 °C/min rate and maintained at this temperature for 5 min. The last interval was performed from 25 to 95 °C at a heat rate of 5 °C/min. These experiments were conducted in duplicate. DSC thermograms allowed the onset, peak and endset temperatures of the thermal transition of starch gelatinization to be determined, with values of 60, 65 and 71 °C, respectively (data not shown).

### 2.3. In Vitro Starch Digestion

#### 2.3.1. In Vitro Potato Starch Digestion Using the *i*-IDS

A previously designed system in our laboratory (*i*-IDS, in vitro intestinal digestion system) (Figure 1) [18] was used for the simulation of the human small intestine. The system consists of two reservoirs. The first one is a jacketed glass vessel (capacity 3 L) used as a feed tank, which is maintained at 37 °C by circulating warm water through the jacket. This tank contained the starch dispersion which was pumped from the feed tank to the mass transfer stage using a peristaltic pump. The second reservoir is a graduated vessel (capacity 25 L) made of high density polyethylene, which was used as a dialysate tank where ultra-purified water was stored at 37 °C and pumped from the dialysate tank to the mass transfer stage using a peristaltic pump. The mass transfer stage consists of a hemodialysis hollow fiber membrane module (Nipro, ELISIO™17H-PP, USA) with a molecular weight cut-off of less than 20 kDa. The feed solution was circulated through the membrane lumen side, while ultra-pure water (dialysate liquid), coming from the dialysate tank, was fed into the shell of the membrane module. The feed flows were defined according to the operating conditions recommended by the supplier.

Three assays were carried out according to three ratios of feed flow (mL/min)/dialysate flow (mL/min): 225/386, 389/386 and 389/248 (nominally named as 250/400, 400/400 and 400/250, respectively), since it is expected that the flow rate influences the mass transfer of reducing sugars obtained from starch digestion. These feed/dialysate flow ratios were set at the same values used in our previous work [18] in order to compare results between studies. Furthermore, the selection of these flow ratios was based on the minimization of the transmembrane pressure. For each experiment, the feed tank was filled with 2 L of feed solution (i.e., gelatinized potato starch dispersion), while the dialysate tank was filled with 19 L of ultra-pure water and 1 L of phosphate buffer solution with continuous stirring at 37 °C, adjusting the pH to 7.0. Prior to the start of the simulation, 6.6 µL of α-amylase was added to the feed vessel. The enzyme was left to act for 1 min while continuing to homogenize, after which the system was put into operation. The enzyme:starch (dry weight basis) ratio was 0.26:1 *v*/*w*, following the procedure performed by Dartois and co-workers [46]. The feed flow circulated through the membrane lumen side, dialysate solution was fed into the shell side of the membrane module, and both tanks were maintained at 37 ± 1 °C during the assays. Each test was carried out for 1 h. In order to determine the reducing sugars generated by the enzymatic digestion of starch and the reducing sugars transferred in the system, simulating the digestive and absorptive functions of the human small intestine, aliquots (2 mL) were taken simultaneously from both the feed and the dialysate tanks during the process time (1 h). The aliquots were taken at intervals of 2 min during the first 20 min of the experiment and then at intervals of 5 min until completing 1 h of assay. In addition, the volume of both tanks was registered during the assays.

#### 2.3.2. In Vitro Potato Starch Digestion Under Batch Conditions

In order to determine the maximum mass of reducing sugars generated by the enzymatic digestion, assays of in vitro starch digestion under batch conditions were carried out. For these studies, a reservoir with identical characteristics to the feed tank (3 L double-walled vessel) was used. Two liters of gelatinized potato starch dispersion (1% *w/v*) at pH equal to 7.0 were prepared in the vessel, following the procedure described above. The vessel was maintained at 37 °C by circulating warm water through the jacket. Prior to the assay, 6.6 µL of α-amylase was added to the vessel, leaving it to act for 1 h min while continuing to homogenize the sample. Two mL aliquots were taken every 2 min during the first 20 min of the experiment, and then at intervals of 5 min until completing 1 h of assay, which were collected to be subjected to measurements of reducing sugars concentration.

All experiments were done in triplicate and results are presented as mean values with standard deviations.

#### 2.3.3. Measurements of Reducing Sugars Concentration

The aliquots obtained from the assays of starch digestion using the *i*-IDS (from the feed and the dialysate tanks) and those obtained from the assays *of* in vitro starch digestion under batch conditions were collected into 3 mL Eppendorf tubes (Mundolab, Santiago, Chile), which contained 20 µL of 1 N hydrochloric acid (Merck, Darmstadt, Germany). These samples were kept for 30 min. At the end of 30 min, the concentration of reducing sugars was measured using Millier’s spectrophotometric method with 3,5-Dinitrosalicylic acid (DNS) [47,48]. For the analysis of the samples, aliquots of 0.5 mL were taken from the Eppendorf tubes and placed in test tubes and mixed with 1.5 mL of DNS solution to be heated for 5 min using a bath of boiling water and then were placed into a cold bath for 3 min. After this process, the samples were diluted with 10 mL of ultra-purified water and the absorbance of the samples was then measured using a spectrophotometer (Shimadzu UV Visible, UVmini-1240, Kyoto, Japan) at 600 nm. To determine the reducing sugars, a calibration curve made with glucose (Sigma Aldrich, St. Louis, MO, USA) was previously performed, allowing the interpolation of the measured absorbance and the calculation of the reducing sugar concentration, expressed as mg glucose equivalent per mL sample.

### 2.4. Dialysis Membrane Process and Phenomenological Approach

In the simulation system, the generation of hydrolytic products from potato starch corresponding to reducing sugars, such as glucose monomers, maltose and dextrins occurs [19,40]. To predict the amount of hydrolytic product formed over time, the reaction kinetics of starch degradation were modeled using the first order equation (Equation (1)) [21]:

(1) $${\mathrm{rs}}_{t}={\mathrm{rs}}_{\infty }\cdot (1-e^{-k\cdot t})$$

where rs_t_ is the reducing sugars mass (g) generated over time, rs∞ is the maximum mass of reducing sugars (g) produced at each operational condition in the *i*-IDS, k is the degradation rate constant (1/min) and t is the time (min). It is relevant to mention that Equation (1) represents a kinetic model of reducing sugar generation.

The amount of reducing sugars generated by starch digestion in relation to the initial potato starch mass can be expressed as a percentage according to Equation (2):

(2) $$\mathrm{Reducing}\;\mathrm{sugars}\;(\%)=\frac{{\mathrm{rs}}_{\infty }}{M_{0}}\cdot 100\%$$

where M_0_ is the initial mass of potato starch (g).

The membrane efficiency is defined as the ability to allow the passage of the hydrolytic products generated during digestion in the *i*-IDS from the feed to the dialysate, which can be quantified in Equation (3) as:

(3) $$\mathrm{Membrane}\;\mathrm{efficiency}\;(\%)=\left(\frac{M_{\mathrm{rsD}}}{M_{\mathrm{rsF}}+M_{\mathrm{rsD}}}\right)\cdot 100\%$$

where M_rsD_ is the dialysate reducing sugars mass (g) and M_rsF_ is the feed reducing sugars mass (g).

The expected overall efficiency of the mass transfer stage in the *i*-IDS with respect to the maximum mass of reducing sugars generated from the batch digestion of starch was determined according to Equation (4):

(4) $$\mathrm{Expected}\;\mathrm{overall}\;\mathrm{efficiency}\;(\%)=\left(\frac{M_{\mathrm{rsD}}}{M_{T}}\right)\cdot 100\%$$

where M_T_ is the maximum mass of reducing sugars generated (g) from batch experiments.

From the experimental results, a phenomenological model is proposed to simulate the reducing sugars transfer process in the in vitro system, based in our previous work [18]. This model is a useful tool for the operation and scaling-up of the *i*-IDS to the human small intestine, including the digestion process of starch. Through the experimental data it became possible to propose a model that allows the concentration of reducing sugars in the feed vessel over time to be known. For the development of the model, the use of the transmembrane pressure values estimated in our previous study [18] was considered.

### 2.5. Statistical Analysis of Data

All experiments were carried out in triplicate using freshly prepared samples, and results are presented as mean values with standard deviations. Analysis of variance was carried out when required using Statgraphic Centurion XVI (version 16.1, Statistical Graphics Corporation, Rockville, MD, USA), including multiple range tests (*p* > 0.05) for separation of least square means.

## 3. Results and Discussion

**Figure 2:**
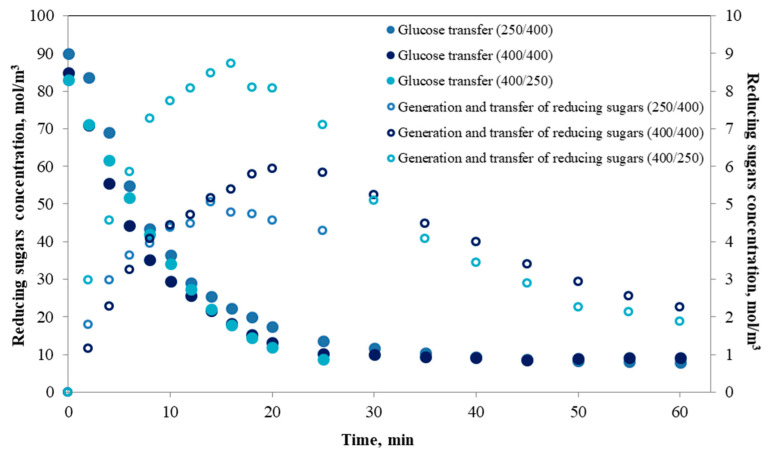
Experimental curves of glucose concentration in the feed tank obtained by Gim-Krumm et al. [7] (primary axis) and concentration of reducing sugars in the feed generated from the enzymatic starch digestion (secondary axis) operating the *i*-IDS at different feed flow/dialysate flow ratios.

In this study, time-concentration curves of reducing sugars in the feed for different operating conditions of feed flow/dialysate flow ratio in the *i*-IDS were obtained (Figure 2). The shape of these time-concentration curves describes the continuous generation of hydrolytic products from starch digestion, together with the corresponding transfer of these products to the dialysate. The increase in concentration of reducing sugars (by the enzymatic digestion of starch) in the feed tank with time occurred until a maximum value was reached, after which a decrease in reducing sugars concentrations as the transfer occurs was noted. The latter was promoted by the operation of the membrane, where the sugars transfer from the feed side to the dialysate side will be determined by the minimum size reached by these molecules, which are capable of penetrating the membrane pores (molecular weight cut-off < 20 kDa). In Figure 2, the experimental results were compared with those previously obtained by our research team [18] in a study of glucose transfer. In this study, the same system was used, where a feed consisting of glucose solution (15 mg/mL) was employed in the *i*-IDS to simulate and extrapolate results of the process of glucose transfer in the human small intestine. The previous study did not consider the effects of other phenomena interacting simultaneously with the sugar transfer process, such as digestion and consequent generation of hydrolytic products. For this reason, the development of the current study was based on a more complex phenomenology, simulating the starch digestion (main source of digestible carbohydrates) in a more realistic manner, which will allow a more detailed understanding of some critical aspects that must be considered when operating new or pre-existing models of human small intestine simulation.

### 3.1. Concentration of Reducing Sugars in the i-IDS under Different Operational Conditions

**Figure 3:**
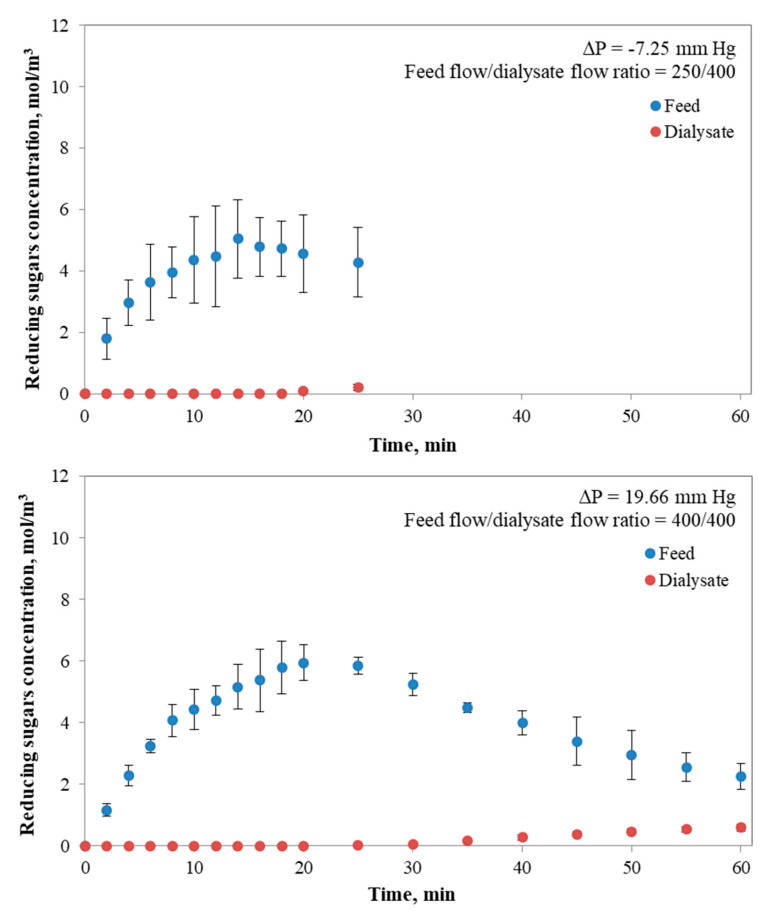

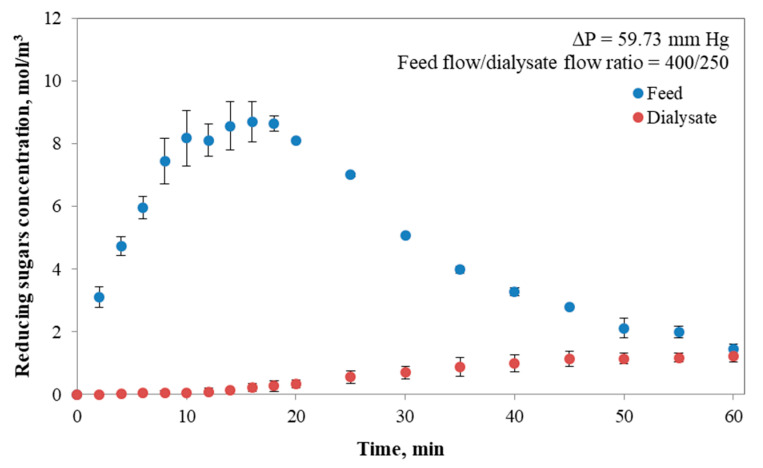
Concentration of reducing sugars in the feed and dialysate tanks over time for different operational conditions of feed flow/dialysate flow ratio and transmembrane pressures.

During starch digestion in the *i*-IDS, the reducing sugars concentration over time in the feed and dialysate tanks for different operational conditions of feed flow/dialysate flow ratio was determined (Figure 3). From the experimental curves, it can be observed that the feed flow/dialysate flow ratio affected the behavior of mass transfer of reducing sugars. Unlike the results previously obtained [18], where the glucose concentration in the feed tank only decreased, the results in Figure 3 show an increase in the concentration of reducing sugars from time 0 until a maximum was reached at a certain time, in function of the operating conditions. From this maximum value, a decrease in concentration begins. The rise in concentration is explained by the generation of reducing sugars, such as glucose, oligosaccharides or dextrins, due to the enzymatic digestion of starch induced by α-amylase. Since the dialysis module is already operating, the transfer of these sugars will be determined by the minimum size that is reached by these molecules. At this point, it should be considered that the analytical measurement included reducing sugars and not directly glucose, as previously described. For the concentration curves, the time at which the inflection point occurs depends on the feed flow/dialysate flow ratio tested (250/400, 400/400 and 400/250). As can be seen from Figure 3, all concentration curves show a delay in the detection of reducing sugars in the dialysate, although there is an evident concentration gradient that promotes the mass transfer between both zones (feed and dialysate). It is known that the most common final products of starch hydrolysis are maltodextrins, glucose, fructose or maltose [36]. However, in the early stages of starch hydrolysis, maltodextrins are generated. These are a mixture of poly- and oligosaccharides with a broad molecular weight distribution. In fact, the molecular weight of commercial maltodextrin with different dextrose equivalent values (2–19) has been found in the range from 9 to 155 kDa [49]. Moreover, results reported by Bednarska (2015) [50] demonstrated that the majority of the products generated after the first 15 min of enzymatic starch hydrolysis had a molecular weight of 70 kDa, as a result of the attack of the enzyme on the accessible linear fragments of high molecular weight amylopectin. For this reason, hydrolysis products with a molecular weight higher than the cut-off of the membrane cannot be transferred to the dialyzed zone, but they can be detected using the DNS method in the feed zone through the reaction with the reducing-end groups of these high molecular weight fractions. In this regard, the membrane behaves as a selective barrier to small molecular weight sugars, just as the human small intestine does.

When the *i*-IDS was operated at a flow ratio of 250/400 (Figure 3), the whole experimental time period (60 min) could not be completed, since when working at a higher flow rate of dialysate compared to the feed flow rate, there is a higher transmembrane pressure on the dialysate side (−7.25 mm Hg). This fact promoted the transfer of water and reducing sugars to the lumen side, causing the overflow of the feed tank in a short period (25 min), and as a consequence, it was necessary to finish the process earlier than anticipated. Furthermore, the convective flow under this condition competes with the diffusive flow, because there is a transfer of reducing sugars across both sides of the membrane, a result that is consistent with those previously observed [18]. However, when the *i*-IDS was operated at a flow ratio of 400/400 (Figure 3), the whole experimental time period was completed. Under this condition, the system tends to equalize the concentrations of reducing sugars in both tanks, tending to an equilibrium state. In turn, the mass transfer rate of hydrolytic products decreases, since the convective and diffusive transfers depend on the concentration gradient between the dialysate and the feed. It was also found under this condition (Figure 3) that the concentration of reducing sugars in the feed tank reaches a maximum, which favors the diffusive mass transfer induced by a higher concentration gradient between the lumen and the shell side of the membrane module, after which this gradient decreases. For this case, the transmembrane pressure is low (19.66 mm Hg), a condition which favors the diffusive mass transfer of reducing sugars in the system.

For the case in which the *i*-IDS was operated at a feed flow/dialysate flow ratio of 400/250 (Figure 3), the reducing sugars transfer was influenced by a diffusive and convective process from the feed side to the dialysate side. Here, the amount of reducing sugars generated from the starch digestion during the testing time was higher in comparison with the other cases studied (see next section). This generated a higher concentration gradient between the lumen and the shell side, and favored the transfer of reducing sugars to the dialysate which is reduced gradually in time, and decreased the rate of reducing sugars transfer to the dialysate to the extent that the concentrations in both tanks are equal. The higher feed flow compared to the dialysate flow generated a pressure difference (transmembrane pressure of 59.73 mm Hg) that favored the mass transfer by convective effect. In this case, the simulated absorption process of reducing sugars turns out to be faster compared to the previous tests, and an equilibrium state for the concentrations between both tanks was reached at ~60 min of testing.

### 3.2. Mass of Reducing Sugars Obtained under Different Operating Conditions of the i-IDS and the In Vitro Batch Digestion of Starch

**Figure 4:**
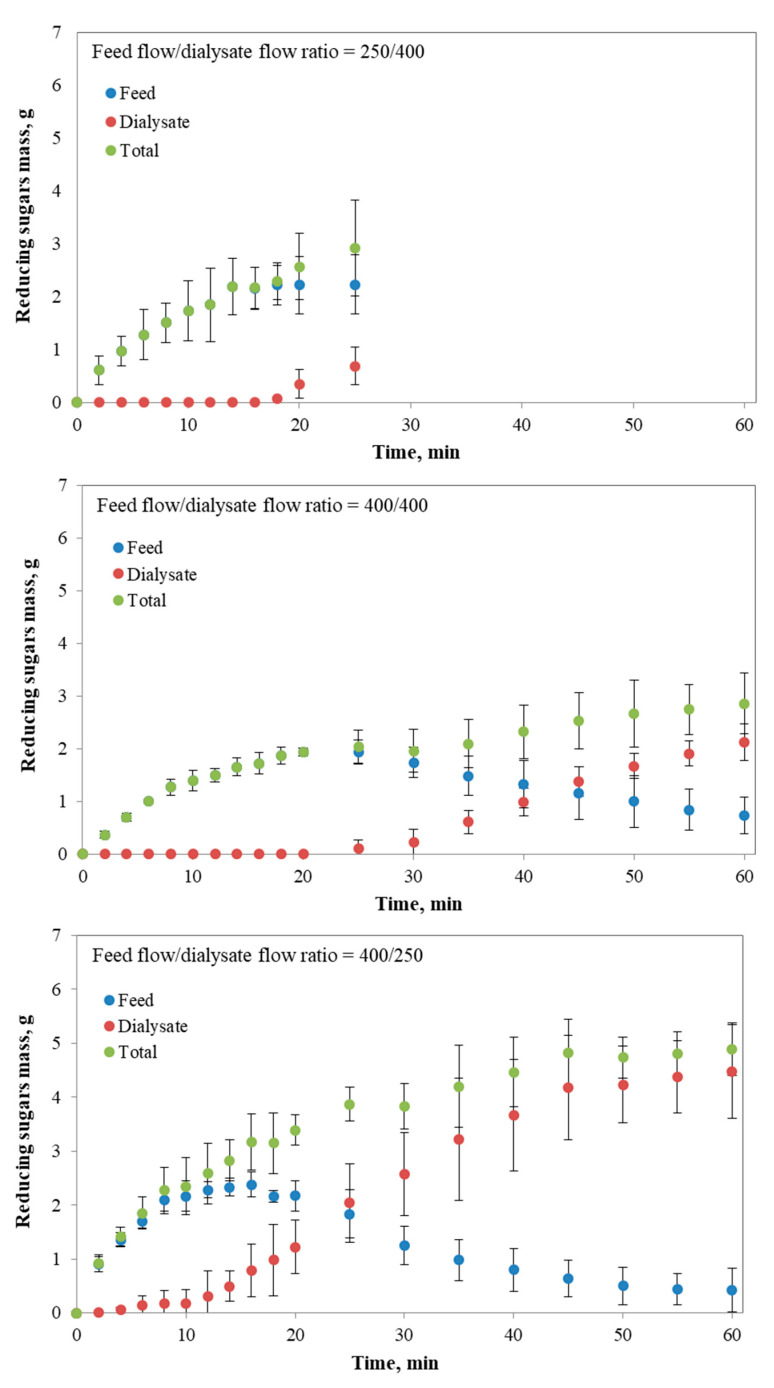

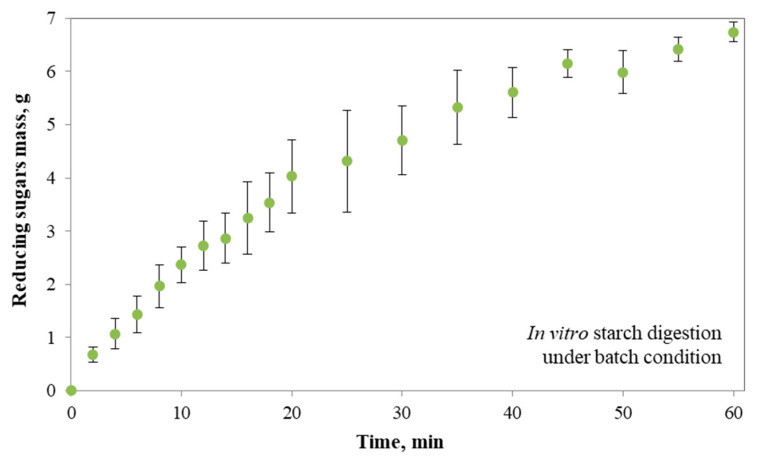
Reducing sugar mass in the feed, dialysate and total over time for different operational conditions of the *i*-IDS and mass of reducing sugars generated under batch condition.

The amounts of hydrolytic products generated during each test, promoted by the in vitro starch hydrolysis and expressed as mass of reducing sugars, for the different operating conditions of feed flow/dialysate flow in the *i*-IDS, and the generation of reducing sugars obtained from the batch digestion of starch are shown in Figure 4.

**Figure 5:**
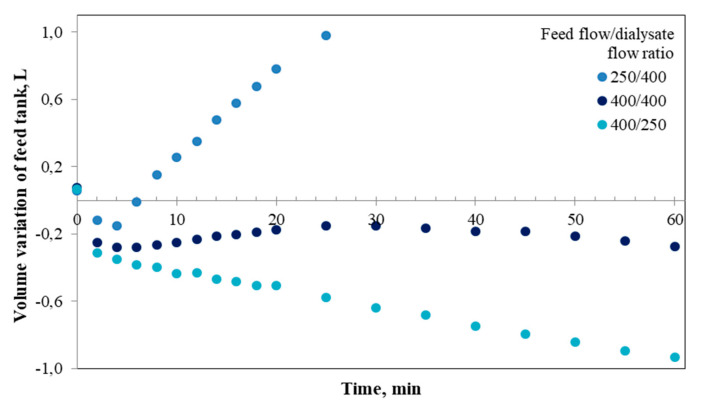
Volume variation of the feed tank, for different operational conditions of the *i*-IDS. Positive values mean accumulation and negative values mean water transfer.

From Figure 4, it can be observed that at the end of the assay (25 min at a ratio of 250/400) ~3 g of reducing sugars were generated. This value is similar to that obtained from the test at a ratio of 400/400 (2.86 g) after 60 min of assay and similar to the sugars mass obtained at 14 min (2.83 g) when the assay was carried out at ratio of 400/250. As previously discussed for the results of concentration, the behavior found in Figure 4 at a ratio of 250/400 can be associated with the differences in pressure between the lumen side and the shell side of the membrane, promoting the transfer of water and reducing sugars to the lumen. The low amount of reducing sugars measured in the dialysate suggests that the transfer of water from the dialysate into the feed could be a factor that affects the passage of hydrolytic products through the membrane. When analyzing the results at an operating ratio of 400/400 (Figure 4), it was observed that the transport of reducing sugars from the feed into the dialysate was initiated at ~25 min, the longest time period for which these sugars were transferred. In any case, and as previously commented, under this operating condition the process is controlled only by a diffusive transport, as found in the human small intestine [27]. Unlike the results mentioned above, the operation of the *i*-IDS at a feed flow/dialysate flow ratio of 400/250 favored the rapid transfer of hydrolytic products into the dialysate, as soon as the generation of reducing sugars begins. Under this condition, a higher amount of reducing sugars generated was obtained (4.9 g), in comparison with the other flow ratios analyzed (2.92 g at 250/400 and 2.86 g at 400/400). This fact can be explained by the higher rate of starch hydrolysis attributed to the prompt transfer of hydrolytic products with molecular weight lower than 20 kDa, since it is known that compounds produced during the starch hydrolysis (e.g., glucose and maltose) can induce uncompetitive inhibition of the catalytic action of α-amylase [1,51]. Alternatively, the rapid starch hydrolysis in the feed tank and subsequent removal of the reducing sugars generated could be related to the higher mass transfer as the feed viscosity reduces, since at low viscosity starch and α-amylase can move freely to contact each other. Furthermore, when the system is operated at a flow ratio of 400/250, it results in a higher loss of water in the feed tank (Figure 5). In consequence, the concentration of substrate and α-amylase in this container is increased, promoting the increase in the rate of starch degradation. This agrees with Michaelis-Menten’s kinetics [1], which describes the rate of generation of hydrolytic products during starch digestion as a function of initial starch concentration. Therefore, given the results in Figure 5, it would be expected that the increase in the concentration of starch and α-amylase in the feed tank would be more significant when the feed flow was higher in comparison with the other operating conditions assayed.

From the in vitro assays of batch starch digestion, the maximum amount of reducing sugars that can be obtained during one hour of hydrolytic reaction with α-amylase under controlled conditions of temperature was determined, with a value equal to 6.7 g (Figure 4). In this study, the degradation rate constant of starch (k) was obtained from the mass generation curve under batch condition and was estimated in 0.037 1/min. This value is in the range of those reported by Goñi and co-workers [21] (k = 0.03 − 0.16 1/min), who applied an in vitro procedure to measure the rate of starch digestion in starchy common foodstuffs, and by Butterworth and co-workers [43] (k = 0.025 − 0.038 1/min), who analyzed the starch amylolysis using plots for first-order kinetics. Now, considering that initial mass of gelatinized starch for all the experiments carried out was of 20 g, it was estimated that the starch hydrolysis obtained at the end of the batch digestion (60 min) was of ~33.5%, based on the 6.7 g of reducing sugars generated and which could be effectively available for absorption in the *i*-IDS. From this value, the expected overall efficiency (%, Equation (4)) of the *i*-IDS was defined, with the purpose of comparing the results obtained from this system with those obtained from the static models commonly used to simulate the in vitro food digestion. When operating the *i*-IDS at feed flow/dialysate flow ratios of 250/400, 400/400 and 400/250, the expected overall efficiencies were 10.2%, 31.5% and 66.4%, respectively (Table 2). These percentages are in accordance with the argumentation previously presented in relation to the impact of the operating conditions on starch hydrolysis and mass transfer of hydrolytic products. However, it highlights the fact that both systems analyzed (i.e., dynamic and static) have marked differences in the results obtained in evaluating the in vitro starch digestion.

The final conversions of starch to reducing sugars in the *i*-IDS were 14.6%, 14.3% and 24.5%, at flow ratios of 250/400, 400/400 and 400/250, respectively (Table 2). The operation of the *i*-IDS at a ratio of 400/250 presented the higher membrane efficiency (91.5%), in line with the explanation given above. On the contrary, when operating at ratios of 250/400 and 400/400, lower membrane efficiencies were obtained due to the lower generation of reducing sugars. With all this in mind, it is important to highlight the impact of the operating conditions of the *i*-IDS, or other similar dialysis membrane-based systems, when studying in vitro digestion of different food matrices, since these conditions determine the diffusive and convective transfers that control the process.

### 3.3. Fouling Analysis and Its Effect on Dialysis Performance

In this study, differences found in the dialysis performance at different operating conditions of the *i*-IDS could also be explained by the potential effect of membrane fouling on the system behavior. Different contents of reducing sugars in the dialysate tank were achieved by absorption and dialysis of hydrolytic products through hollow fiber membranes in the *i*-IDS. From the experimental results, it was established that different phenomena determine the behavior of the system. Therefore, in order to understand in a better way the response of the variation of reducing sugars concentration in the feed with time, a mathematical modeling was performed adopting the realization of a mass balance based on our previous work [18].

The transfer phenomena of reducing sugars by convective and diffusive effects from the feed tank to the dialysate tank can be represented by the following expression (Equation (5)):

J_rs_ = J_D_ + J_C_ (5)

where J_rs_ is the total flux of reducing sugars in the dialysis membrane process (g/m^2^ min), J_D_ is the flux of reducing sugars transferred by diffusion (g/m^2^ min), and J_C_ is the flux of reducing sugars transferred by convection (g/m^2^ min).

The overall mass transfer of reducing sugars through the membrane (N_rs_, g/ min) can be described as the difference of concentration (diffusive or osmotic effect) and transmembrane pressure (convective or hydrostatic effect) through the Equation (6) [18]:

(6) $$N_{\mathrm{rs}}=({\mathrm{KA}}_{T}+K_{\mathrm{UF}}\;\Delta P)\cdot \left(C_{F}^{\mathrm{rs}}-C_{D}^{\mathrm{rs}}\right)$$

where K is the overall mass transfer coefficient (m/min), A_T_ is the total area of mass transfer (m^2^), K_UF_ is the ultrafiltration coefficient reported by the supplier of the dialysis membrane modules (m^3^/Pa min), ΔP is the transmembrane pressure (Pa), $C_{F}^{\mathrm{rs}}$ is the concentration of reducing sugars in the bulk of the feed (g/m^3^), and $C_{D}^{\mathrm{rs}}$ is the concentration of reducing sugars in the bulk of the dialysate (g/m^3^).

The mathematical model proposed previously [18], which represents the process of reducing sugars transfer in the membrane system, was described in Equation (7) as:

(7) $$\ln\left(\frac{C_{F}^{\mathrm{rs}}-C_{D}^{\mathrm{rs}}}{C_{\mathrm{rs}}^{0}-C_{D}^{\mathrm{rs}}}\right)=-({\mathrm{KA}}_{T}+K_{\mathrm{UF}}\;\Delta P)\cdot \frac{t}{V_{F}}$$

where $C_{\mathrm{rs}}^{0}$ is the initial reducing sugars concentration (g/m^3^) and V_F_ is the feed volume (m^3^). This last equation has been taken as a good starting point for modeling the results obtained in this study, considering the incorporation of the kinetics of starch digestion (Equation (1)) and eliminating the term $C_{\mathrm{rs}}^{0}$, since in this case this value was equal to 0, which increases gradually over time.

Following the approach mentioned above, the hydrolytic products in the system will depend on the kinetics of reducing sugars generation, together with the diffusive and convective effect. This means that the mass transferred through the membrane (N_rs_) is equal to the mass of reducing sugars generated over time, which can be obtained by deriving the kinetic equation (Equation (1)). Therefore, this mass balance can be expressed by the following Equation (8):

(8) $$\frac{{\mathrm{drs}}_{t}}{\mathrm{dt}}=N_{\mathrm{rs}}=\left({\mathrm{KA}}_{T}+K_{\mathrm{UF}}\Delta P\right)\cdot \left(C_{F}^{\mathrm{rs}}-C_{D}^{\mathrm{rs}}\right)$$

By discretizing Equation (8), it is possible to determine the concentration of reducing sugars in the feed $\left(C_{F}^{\mathrm{rs}}\right)$ in function of time (Equation (9)). The mass of reducing sugars generated over time (rs_t_ and rs_t−1_) by the in vitro starch digestion can be determined from the generation of hydrolytic products (Equation (1)).

(9) $$C_{F}^{\mathrm{rs}}=\frac{{\mathrm{rs}}_{t}-{\mathrm{rs}}_{t-1}}{\Delta t}\cdot \frac{1}{\left(K_{\mathrm{UF}}\cdot \Delta P\right)+\left(K\cdot A_{T}\right)}+C_{D}^{\mathrm{rs}}$$

where rs_t−1_ corresponds to the moles of reducing sugars generated in the previous time period (g), and Δt is the time step (min).

Certain operational variables can impact membrane separation of poly- and oligosaccharides, affecting in turn the overall efficiency of the process [52]. The membrane fouling often causes dramatic decreases of the flux. This explains the fact by which the membrane fouling should be considered when studying its implications for the separation efficiency of the process. With this in mind, in this study a parameter of mass transfer resistance (R_MT_, dimensionless) was proposed, which represents the formation and degradation of a dynamic fouling layer that decreases the mass flux over time through the membrane. In this context, Equation (8) can be modified by incorporating the term of mass transfer resistance, according to Equation (10):

(10) $$\frac{{\mathrm{drs}}_{t}}{\mathrm{dt}}=N_{\mathrm{rs}}=\frac{\left({\mathrm{KA}}_{T}+K_{\mathrm{UF}}\Delta P\right)}{R_{\mathrm{MT}}}\cdot \left(C_{F}^{\mathrm{rs}}-C_{D}^{\mathrm{rs}}\right)$$

In the current study, the membrane fouling could be due to (i) solid impurities from the commercial starch, deposited on the membrane, (ii) formation of a biopolymer layer (i.e., composed by undigested starch and reducing sugars generated by enzymatic hydrolysis, whose sizes are larger than ~20 kDa) at the membrane surface and/or (iii) the gelatinized starch itself filling the pores of the membrane [53]. This fact is in line with reported in vivo observations of gastrointestinal mucoadhesion. For example, some studies, which focused on drug bioavailability, indicate that, by adhering to the mucus layer of the gastrointestinal tract, mucoadhesives induce rapid drug absorption. However, mucoadhesion can be significantly limited by the constant passage of food or by colloidal particulate systems, which generates an unavoidable interaction leading to fouling on the mucous gel layer [54,55]. Due to this, the study of intestinal absorption using simplified models based on membranes that act as an epithelial barrier has promoted new research lines focused on improving nutrient bioavailability. In fact, different chemical modifications of synthetic membranes (e.g., cell cultures-based membrane) have been studied, since it has been recognized that several constituents lead to polymer membrane fouling, such as dissolved organic/inorganic compounds, colloids, cells and suspended solids [56].

Thus, the fouling for the dialysis process in the *i*-IDS can be considered as a dynamic phenomenon, since the hydrolytic products from the starch reaction vary with time, and also material adhering on the membrane surface can be removed during the process time. In consequence, the value of R_MT_ for each operating condition can be determined by rewriting Equation (10) as follows (Equations (11) and (12)):

(11) $$C_{F}^{\mathrm{rs}}=\left(\frac{{\mathrm{rs}}_{t}-{\mathrm{rs}}_{t-1}}{\Delta t}\cdot \frac{1}{\frac{\left(K_{\mathrm{UF}}\cdot \Delta P\right)+\left(K\cdot A_{T}\right)}{R_{\mathrm{MT}}}}\right){+C}_{D}^{\mathrm{rs}}$$

Thus,

(12) $$R_{\mathrm{MT}}=(C_{D}^{\mathrm{rs}}-C_{F}^{\mathrm{rs}})\cdot \frac{\Delta t}{{\mathrm{rs}}_{t}-{\mathrm{rs}}_{t-1}}\cdot [\left(K_{\mathrm{UF}}\cdot \Delta P\right)+\left(K\cdot A_{T}\right)]$$

**Figure 6:**
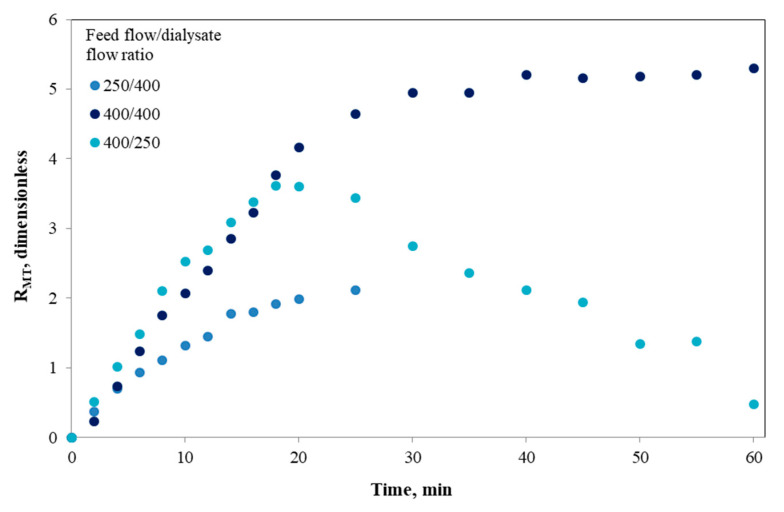
Variation of the mass transfer resistance (R_MT_) with time, for different operational conditions of the *i*-IDS.

In Equation (12), the mass transfer coefficient (K) and ultrafiltration coefficient (K_UF_) were estimated using the same mathematical methodology described by Gim-Krumm and co-workers [18]. As it can be expected, and in agreement with the results of concentration and mass of reducing sugars generated from the in vitro starch digestion in the *i*-IDS discussed in the Section 3.1 and Section 3.2, respectively, the value of R_MT_ was a function of time and of the operating conditions of the *i*-IDS (Figure 6). First of all, this highlights the fact that for the three feed flow/dialysate flow ratios analyzed, a mass transfer resistance was observed, which occurred immediately after starting the *i*-IDS operation. It is known that fouling can occur in a variety of membrane systems after a few minutes of operation [57]. However, this did not occur in our previous study [18], since we only worked with a glucose solution. Therefore, the glucose (size ~180 Da) was completely available for the transfer process, since there was a high concentration gradient, which promoted mass transfer in the system without being altered by some interference phenomenon. Nevertheless, in the current study, a more complex matrix based on starch was digested in the *i-*IDS, where the process becomes dependent on the generation rate of reducing sugars for their subsequent absorption. In turn, the operating conditions influenced the loss or gain of water in both sides of the system, which can impact the formation of large hydrolytic products that favor membrane fouling for the dialysis process [58,59,60].

When the *i*-IDS was operated at a flow ratio of 250/400 (Figure 6), an increase of the values of R_MT_ with time was obtained, and the lowest values after 25 min of assay, when comparing with the other operating conditions, were reached. This last behavior can be associated with the continuous inflow of water from the dialysate to the feed, which generates a cleaning effect of the fouling layer on the membrane surface. However, at a ratio of 400/250, it was possible to observe an increase in the mass transfer resistance over time up to 20 min of operation reaching a maximum. Then, this resistance decreases gradually over time, which reduces the fouling effect and favors the mass transfer to the dialysate. These increases in mass transfer are also favored by the flow of water from the feed to the dialysate, facilitating the transport of hydrolytic products with sizes lower than 20 kDa. Moreover, for the assay performed at a ratio of 400/400, the resistance values were similar to those obtained at a ratio of 400/250 up to 18 min, and then, R_MT_ continued to rise up to ~30 min of assay, after which it was held constant throughout the experiment. For this condition, the evolution curve of R_MT_ shows an inflection point close to 25 min, a time that coincides with the beginning of the mass transfer of reducing sugars to the dialysate. In addition, at an operating ratio of 400/400, not only does fouling formation occur, but also, in comparison with the other conditions, there is no significant flow of water between the feed and the dialysate in the system (Figure 5). This minimizes the interference to the mass transfer towards the dialysate, so there is an additional effect on the mass transfer resistance associated with the transmembrane pressure that turned out to be similar.

### 3.4. Scaling-Up to the Human Small Intestine

The experimental results of reducing sugars absorption of this study were extrapolated to those found in the human small intestine. For this purpose, the phenomenological model described by Equation (11) and the overall mass balance of the system were used as follows: (i) the mass of reducing sugars transferred in the human small intestine was used to estimate an expected efficiency in the *i*-IDS, (ii) the overall mass balance and the expected efficiency allowed a final concentration of reducing sugars in the feed solution to be determined, and (iii) by using the above concentration of reducing sugars and Equation (11), the operation time needed to reach the same mass of reducing sugars transferred in the *i*-IDS was computed for each operating condition. This approach implies that the absorption process of any nutrient towards the human small intestine can be represented using the *i*-IDS, according to fixed experimental parameters. For this analysis, glucose transfer in the small intestine was used as a reference, since predicting and controlling the glucose absorption due to the ingestion of starchy food is of great interest worldwide. Furthermore, the most widely used method for estimating the kinetics of in vitro starch digestion consists of simulating gastrointestinal conditions in order to measure the glucose release at different times [1,61].

The results of scaling-up the *i*-IDS to the human small intestine are shown in Table 3. The operating time in the *i*-IDS required to reach the mass absorbed of reducing sugars in the human small intestine (1.06 g) ranged between 30 and 64 min, according to the feed flow/dialysate flow ratio operated in the system. These time values are lower than those found in the human small intestine (180 min), according to literature data [27,53]. These differences are mainly associated with the higher feed flows used in the *i*-IDS (250–400 mL/min) with respect to intestinal flow (3 mL/min [62,63,64]) and also with the lower area of mass transfer (1.7 m^2^ vs. 30 m^2^ [65]). In our previous work using the *i*-IDS [18], glucose absorption, understood as glucose transfer, was reached at lower times (2.1–2.7 min at similar feed flows), which was mainly explained by the fact that the system had a high concentration of glucose from the beginning of assay and no hydrolytic reaction was involved (i.e., system without digestion). For this reason, there was no time period that would generate a product and therefore increase the total process time. Neither was there a high concentration gradient from 0 min, by which the mass transfer immediately began. On the contrary, and for this case study, a longer time of mass transfer was obtained because of the time required by the enzyme to hydrolyze the gelatinized starch in the feed.

By operating the *i*-IDS, it is possible to study the generation and absorption of hydrolytic products obtained from diverse starchy food matrices and to scale-up the results to the human small intestine using Equation (11). The extrapolation to the human small intestine will require the use of an initial concentration of starch ($C_{F\;\mathrm{starch}}^{0}$), such that the amount of products generated (rs_t_) be greater compared to the flow of products transferred towards the intestine (N_rs_), considering that the human small intestine is capable of transferring ~96% [66] of the hydrolytic products generated from the digestion of starch. However, the scaling-up results obtained here are limited to the operating conditions used. Hence, the testing time (maximum 25 min for the assay at 250/400 and 60 min at ratios of 400/400 and 400/250), the maximum generation of products for each feed flow/dialysate flow ratio and the mass transfer resistance (R_MT_) can effectively describe the experimental behavior under these considerations. However, it should be noted that the mathematical modeling can also be used for longer processing times than those used in this approach.

The mathematical model proposed includes real phenomena occurring in the human small intestine, such as osmotic and hydrostatic transport. Therefore, this simple model could be used to simulate and understand, when testing in vitro systems, the complex behavior of the human small intestine.

In summary, the *i*-IDS can be used to study food digestion and absorption, using similar feed flows and flows ratios as tested here, if the dialysis membrane module is the same. When using a different dialysis membrane module and different flows, the evaluation of results can be performed using the phenomenological model proposed. Thus, the times resulting from a different food matrix in a different laboratory prototype can be related to the real operation of the human small intestine by using the mathematical modeling and scaling-up methodology here proposed.

Finally, it is proper to point out that the *i*-IDS is a versatile system, which makes it suitable for hypothesis building and hypothesis testing when studying digestion and absorption processes in the human small intestine of different starch-based food matrices. The main advantage of the *i*-IDS is the possibility that hydrolytic products generated from digestion can be selectively removed during the digestion process (i.e., absorption). Nevertheless, the formation of membrane fouling during the separation processes needs further studies to propose a phenomenological interpretation with respect to the physiological behavior. In fact, it has been recognized that effective human intestinal permeability of rapidly absorbed compounds in vivo (e.g., glucose) is mainly determined by the membrane permeability, which it implies that the membrane of the intestinal mucosa is the main diffusion barrier for both passively and actively absorbed solutes [69]. On this basis, an additional effort must be made for a next improvement step of the *i*-IDS where membrane fouling can be controlled and interpreted. In addition, this model has the challenge of incorporating physiological events that mimic the functioning of the human small intestine. For the latter, a good starting point will be the application of the new standardized protocol developed by the COST INFOGEST network, where physiological intestinal conditions such as electrolytes incorporation, enzyme activities, bile, dilution, pH and time of digestion will be simulated [70]. In addition, the *i*-IDS could also be used as an exploratory system for studying and understanding digestion and absorption processes using other food matrices. However, further studies using this model are required if food matrices based on fat, proteins or more complex carbohydrates are of interest for testing.

## 4. Conclusions

This work deepens our understanding of starch digestion and the consequent absorption process of the hydrolytic products generated, as found in the human small intestine. By carrying out assays of in vitro digestion with α-amylase, gelatinized starch dispersions were digested in an in vitro intestinal digestion system (*i*-IDS) based on a hollow fiber dialysis membrane process. This study is innovative with respect to previous research conducted by our research team, because it considers the impact of simultaneous digestion and absorption processes occurring during intestinal digestion of starchy foods and adopts phenomenological models that deal in a more realistic manner with the behavior found in the human small intestine.

From the operation of the *i*-IDS at different flow/dialysate flow ratios, different curves were obtained for the generation and transfer of reducing sugars. This demonstrates that the operating conditions affected the behavior of mass transfer of reducing sugars because of diffusive and convective effects. However, the mass transfer behavior was also greatly affected by membrane fouling, a dynamic phenomenon occurring in the *i*-IDS, which was observed for all the experimental conditions tested. Finally, the experimental results obtained were extrapolated to those found in the human small intestine, where the times reached to transfer the hydrolytic products ranged between 30 and 64 min, according to the flow ratio used.

The *i*-IDS is a versatile system that can be used to predict digestion and absorption behaviors in the human small intestine of different starch-based food matrices, but could also be used as an exploratory system for studying and understanding these processes using other food matrices based on fat, proteins or more complex carbohydrates. However, further studies using the *i*-IDS are required to control and interpret membrane fouling, and also to simulate relevant physiological conditions as found in the human small intestine.

## Figures and Tables

**Table 1:** Composition of the potato starch (g per 100 g of dry sample weight), according to information given by the manufacturer.

Energy Content (kcal)	314
Protein	0.2
Total fat	0.1
Total carbohydrate	82.6
Sodium	0.0076

**Table 2:** Percentages of efficiency of the in vitro intestinal digestion system (*i*-IDS).

Feed Flow/Dialysate Flow Ratio	Starch Digested (%)	Membrane Efficiency (%)	Expected Overall Efficiency (%)
250/400	14.6	23.6	10.2
400/400	14.3	74.4	31.5
400/250	24.5	91.5	66.4

**Table 3:** Scaling-up results of the *i*-IDS to the human small intestine.

Parameter	Human Small Intestine	Feed Flow/Dialysate Flow Ratio		
		250/400	400/400	400/250
Reducing sugars transfer, %	96.4 ^1^	24.1	24.1	24.1
$C_{F\;\mathrm{starch}}^{0}$, mg/mL	2.2	2.2	2.2	2.2
$C_{F\;\mathrm{starch}}^{t}$, mg/mL	0.079	0.50	0.62	0.37
$C_{D}^{\mathrm{rs}}$, mg/mL	0	0.05 ^2^	0.05 ^2^	0.05 ^2^
${\mathrm{rs}}_{t}$, g	-	3.41	3.17	3.66
V_F_, L	0.5 ^3^	2	2	2
N_rs_, g	1.06	1.06	1.06	1.06
A_T_, m^2^	30 ^4^	1.7	1.7	1.7
ΔP, mmHg	-	−7.25 ^5^	19.66 ^5^	59.73 ^5^
$C_{F}^{\mathrm{rs}}$, mg/mL	-	1.2	1.1	1.3
Feed flow, mL/min	3 ^6^	250	400	400
Time, min	180 ^7^	30	64	30
R_MT_	-	2.3	5.5	3

^1^ Based on the value of glucose transfer reported in literature [66]. ^2^ Mean value obtained for concentrations of reducing sugars in the dialysate for all the experimental runs. ^3^ The stomach volume was defined as a mean value from literature data [6,67,68]. ^4^ Value of the small intestine transfer area reported in literature [65]. ^5^ Based on the value reported by Gim-Krumm and co-workers (2018) [18]. ^6^ Value of the flow fed into the human small intestine reported in literature [62,63,64]. ^7^ Intestinal digestion time based on literature [27,53].

## Nomenclature

ΔP	Transmembrane pressure (Pa)
A_T_	Total area of mass transfer (m^2^)
$C_{\mathrm{rs}}^{0}$	Initial reducing sugars concentration (g/m^3^)
$C_{F\;\mathrm{starch}}^{0}$	Initial starch concentration in the bulk of the feed (g/m^3^)
$C_{D}^{\mathrm{rs}}$	Concentration of reducing sugars in the bulk of the dialysate (g/m^3^)
$C_{F}^{\mathrm{rs}}$	Concentration of reducing sugars in the bulk of the feed (g/m^3^)
$C_{F\;\mathrm{starch}}^{t}$	Concentration of starch in the bulk of the feed over time (g/m^3^)
J_C_	Flux of reducing sugars transferred by convective effect (g/m^2^ min)
J_D_	Flux of reducing sugars transferred by diffusive effect (g/m^2^ min)
J_rs_	Total flux of reducing sugars in the dialysis membrane process (g/m^2^ min)
K	Overall mass transfer coefficient (m/min)
K_UF_	Ultrafiltration coefficient (m^3^/Pa min)
k	Degradation rate constant (1/min)
M_0_	Initial mass of potato starch (g)
M_rsD_	Dialysate reducing sugars mass (g)
M_rsF_	Feed reducing sugars mass (g)
M_T_	Maximum mass of reducing sugars generated from batch experiments (g)
N_rs_	Overall mass transfer of reducing sugars transferred through the membrane (g/min)
R_MT_	Mass transfer resistance (dimensionless)
rs_∝_	Maximum mass of reducing sugars (g)
rs_t_	Mass of reducing sugars generated over time (g)
t	Time (min)
V_F_	Feed volume (m^3^)
